# Emergence of collective behaviours from local Voronoi topological perception

**DOI:** 10.1098/rsos.231537

**Published:** 2024-06-05

**Authors:** Ivan Gonzalez, Jack Tisdell, Rustum Choksi, Jean-Christophe Nave

**Affiliations:** ^1^ Department of Mathematics and Statistics, McGill University, Montreal, Quebec, Canada

**Keywords:** collective behaviours, Voronoi diagram, Delaunay topology, milling, queuing

## Abstract

This article addresses how diverse collective behaviours arise from simple and realistic decisions made entirely at the level of each agent’s personal space in the sense of the Voronoi diagram. We present a discrete-time model in two dimensions in which individual agents are aware of their local Voronoi environment and may seek static target locations. In particular, agents only communicate directly with their Voronoi neighbours and make decisions based on the geometry of their own Voronoi cells. With two effective control parameters, it is shown numerically to capture a wide range of collective behaviours in different scenarios. Further, we show that the Voronoi topology facilitates the computation of several novel observables for quantifying discrete collective behaviours. These observables are applicable to all agent-based models and to empirical data.

## 1. Introduction

The connection between individual and collective behaviour in biological systems has fascinated researchers for decades. A well-studied paradigm entails the tendency of groups of individual agents to form flocks, swarms, herds, schools, etc. As we discuss further in §1.1, many mathematical models from discrete to continuum have been presented and studied to capture the emergence of collective behaviours from postulated local laws. These models comprise components—for example, averaging orientation directions with Euclidean distance weights to capture alignment, or phenomenological interaction potentials (kernels) for repulsion/attraction—which in addition to facilitating numerical computations, lend themselves well to formal, rigorous, or multi-scale mathematical analysis.

**Figure 1.**
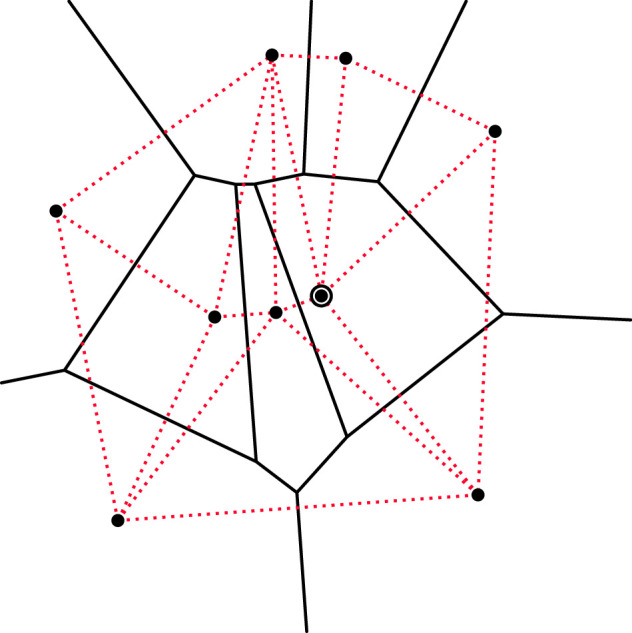
A Voronoi diagram and dual graph. The Voronoi diagram generated by a set of points, consisting of the solid bordered regions, and its dual graph (dotted red) offer a natural communication topology for agent-based models and also give rise to many broadly applicable observables. The Voronoi (dual) topology differs from other communication networks—in particular, k -nearest neighbour—in several respects. For example, focusing on the encircled site, its *second-nearest* site is not among its Voronoi neighbours at all. Moreover, different sites generally have different numbers of Voronoi neighbours.

Here, we take a different approach, divorced from any underlying goal/bias for the potential mathematical analysis of the model. We directly address what we believe to be an important and useful question in the modelling of collective behaviour: how do collective behaviours emerge from simple and realistic decisions made entirely at the level of the individual’s personal space? We argue that the Voronoi diagram provides that personal space. Hence, our underlying assumption is that agents base their decisions on their Voronoi cell and the behaviours of their immediate Voronoi-neighbouring agents. Such neighbouring agents are simply those whose personal space is adjacent to that of the given individual. An example of Voronoi diagram is shown in figure 1 along with its dual graph.

Based solely on the topology this neighbouring connectivity induces, we present a movement scheme (a velocity) via a synthesis (i.e. a weighting) of three competing tendencies: repulsion from the closest neighbour, homing towards a target (or targets) and alignment with the directions of neighbouring agents. This movement scheme is the basis for our model which we call Voronoi topological perception (VTP). While other models are also based upon similar three tendencies, and several have components using the Voronoi topology, ours is distinct in that it is entirely based upon the geometry of an agent’s (Voronoi) personal space. To discuss further the scope and novelty of VTP, we briefly review some of the main modelling paradigms for collective behaviours, and the resulting large body of literature.

### 1.1. Overview of current models

We first present three influential models achieving coherent behaviour solely through symmetric alignment interactions. Vicsek *et al.* [1] introduced a simple kinematic model, where, amid random noise, a transition to ordered behaviour is obtained by averaging over the velocities of neighbours that fall within a metrically finite region, see [2] for analysis. Later, Cucker and Smale [3] introduced a flocking model (C-S) that, in contrast with Vicsek’s, considers a global interaction where each agent is influenced by every other individual. Consequently, C-S presents conservation laws that, on one hand, fix the regimes through the initial conditions as for some physical system (e.g. thermodynamical) but, on the other, seem unreasonable for systems of active, decision-making individuals. Another issue, pointed out by Motsch and Tadmor [4] is that C-S invalidates the dynamics of small sub-flocks at long range; this problem is addressed in their model (M-T). Precisely, M-T introduces the notions of active sets to quantize the neighbour’s influence as well as the notion of relative distances. The latter being supported by the experiments on bird flocks due to Ballerini *et al.* [5] demonstrating many flocking behaviours to be density invariant; i.e. where the behaviour is essentially unchanged as a given configuration of interacting agents scales in (spatial) size. As we will see, a (distinct) notion of relative distance is a direct consequence of our topological perception framework. Note these three approaches do not, in general, produce regimes other than velocity coherence. In this regard, much adapting has been done to produce aggregation and other biologically accurate behaviours by means of long-range attraction, short-range repulsion as well as hierarchy and leadership effects, see [6–12]. Other interesting variants include incorporating: (i) limited peripheral view [4]; (ii) time delays accounting for limited processing aptitudes [13,14]; and (iii) active and passive distinction of agents [15–19]. Other important kinematic approaches which produce rolling and milling behaviours similar to ours are models of d’Orsogna *et al.* [20] and Bernoff-Topaz [21,22], which consider attraction and repulsion through potential as well as exogenous forces. The reader is also referred to seminal work done by Mogilner and Edelstein-Keshet *et al.* in the matter of modelling interactions through the potential formulation [23–25].

Particularly relevant to our approach is a family of models known as *zone-based* that generalize Vicsek’s. Precisely, endogenous interactions act over non-overlapping concentric regions. Among this vast family, one finds the popular boids model introduced by Reynolds in 1987 [26], the Huth and Wissel model of homogeneous fish schools [27], a recent approach by Bernardi and Scianna (B-S) in [28] as well as the seminal Couzin model [29] with hierarchies between the different interactions; the Couzin model was later used in the context of effective leadership and propagation of directional awareness in [30].

Importantly, the *zone-based* framework has been shown to agree with real-life data, for example, Lukeman *et al*. [31] discuss how the dynamics of surf scoters (*Melanitta perspicillata*) can be accurately described by different models in this family after an optimal fit of their parameters. We point out that many zone-based interactions are often realized as gradients of artificial potentials (although qualitative features often do not depend on the precise form of such potentials, e.g. [32]), and this approach is seen in biological models as well as implemented in multi-agent control systems as in [33]. Furthermore, these approaches often involve steering towards the centre of mass of a possibly large number of agents, which is appropriate for automated multi-agent control but not so realistic for biological species with limited processing capabilities.

Olfati-Saber and others have worked to present very broadly applicable theoretical frameworks for flocking in multi-agent systems in [34–36], especially for the case of linear dynamics (in both continuous and discrete time).

The ‘social force’ pedestrian model (H-M) from Helbing and Molnár [37] (see also the seminal work [38]) strives for a realistic human pedestrian flow without using a density-invariant communication notion; i.e. behaviours are considerably altered as a given configuration of interacting agents gets clustered or spread out. For a comprehensive summary of progress made in the realm of pedestrian dynamics from both macroscopic and microscopic scales, the reader is referred to Chraibi *et al.* [39]. We remark that, depending on the context, it is a model’s prerogative to be described in terms of accelerations or velocities: authors can choose to encode (or not) the fact that cars or heavy multi-agent systems closely follow an inertial Newton-type behaviour while pedestrians and other biological species can accelerate and brake almost instantaneously—thus, do not generally think in terms of accelerations at the tactical level. While this ‘convention’ is natural, many successful models do not adapt to it; e.g. H-M is a pedestrian model based on acceleration. H-M and other knowledge-based human pedestrian models stand in contrast with comparatively recent deep learning approaches. This dichotomy is explored in detail in the review article [40]. The follow-up [41] gives a broad overview of continuous time pedestrian models including various approaches and ranging in their mathematical sophistication.

Finally, we emphasize that others have previously used Voronoi diagrams in multi-agent models and control systems, and they feature prominently in the literature on epithelial and biological tissues [42,43]. Ginelli and Chaté [44], inspired by [5], show that adapting Vicsek’s model to use a Voronoi communication topology produces qualitatively novel behaviours—here and throughout, a ‘communication topology’ is simply the graph that determines who influences whom at a given moment of the dynamics. Grégoire and Chaté [10] describe a minimal extension of [44], which achieves selected coherent behaviours despite ‘unfavourable conditions’. Following the study of Ballerini *et al.* [5] on comparing the communication topologies induced by metric distance versus k -nearest neighbours, the Couzin model has also been adapted by Kolpas *et al.* [45] to use the Voronoi diagram (and its dual graph) as a proxy to the k -nearest neighbour topology. We remark that the k -nearest and the Voronoi topology are generally different graphs since the kth closest neighbour does not need to be a Voronoi neighbour (for k≥2 ), and, conversely, an agent may have more than k Voronoi neighbours (see figure 1).

Where the above models use the Voronoi topology, the multi-vehicle control system developed by Lindhe *et al*. [46] considers a limited range of neighbours, as Vicsek, but from these, constructs a Voronoi region whose geometry influences the control. We remark that Strandburg-Peshkin *et al*. [47] show that Voronoi-based models empirically outperform metrical and k -nearest neighbour-based models in the sense of information propagation through the network, at least in regimes which admit fair comparison by their methods.

### 1.2. Purpose and scope of our work

First off, we do not claim that VTP is an improvement over any previous model. We are providing a new model from the microscopic perspective (as opposed to thermodynamical/macroperspective), described in terms of velocities (as opposed to acceleration and other inertial terms), and within the ‘school’ of Voronoi topology-induced regions of influence (as opposed to metric regions or k -nearest influence).

The model adhering to these three categories that would be closest to ours [45] presents key differences: (i) its repulsion component is an average while ours is simpler and swift; (ii) its repulsion and alignment are hierarchical while ours can take effect simultaneously; and, more importantly, (iii) our method not only uses the Voronoi topology but also gauges the geometry and ‘size’ of the personal space to adjust the speed rather than assigning a constant value. Moreover, to keep listing fundamental properties, our framework limits some of the assumptions made on the population when compared with other models from §1.1: (iv) agents are not required to steer towards centres of mass nor perform complex averaging of non-unitary vectors (more in §2.1.2). (v) We do not assume long-range attraction or reorientation where agents need to be aware of all other agents at all times; instead, agents are aware of only a small number of neighbours, and, through the non-locality of the Voronoi diagram, information from far away does require several time steps to reach an agent. This reduced number of neighbours in the communication topology leads VTP to benefit from a notion of *relative distance* analogous to [4] (see §2.1.2).

**Figure 2.**
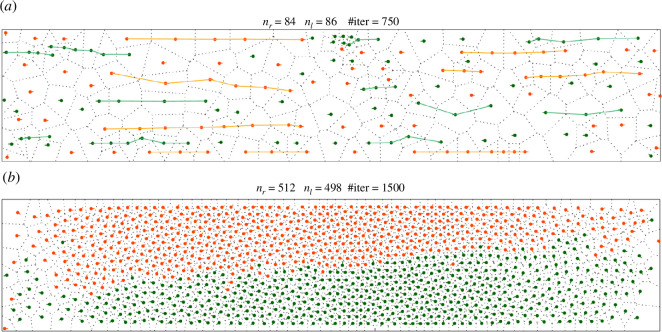
Emerging behaviours in the bidirectional corridor (see §4). Agents *X_r_* moving to the right are shown in orange and *X_l_* moving to the left in green: (*a, click here to view simulation*) Regime I shows significant amounts of queuing. The queuing structure (graph) Ξ*_r_* is displayed in orange and Ξ*_l_* in green; and (*b, click here to view simulation*) Regime V shows the two subpopulations separated by a long interface and ‘sliding’ along each other.

**Figure 3.**
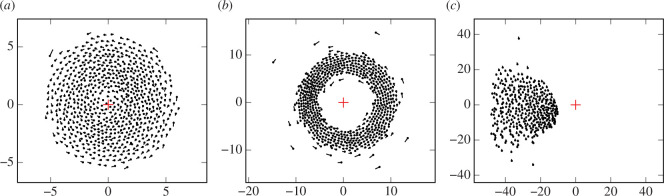
Emerging behaviours for a single point target in the plane under Model II with *n* = 700 agents (see §3). From the left, (*a*, click to run simulation) pinwheel *ν* = 3, (*b*, click to run simulation) ring *ν* = 13 and (*c*, click to run simulation) aligned orbiting cluster *ν* = 40. The red crosshair indicates the target point in each figure.

We view our model—that is our scheme for synthesizing repulsion, homing and attraction—as on one hand, rather simple and easy to implement with only two effective parameters, and on the other hand, complex enough to exhibit a spectrum of behaviours in different scenarios. Note that the literature has innumerably many models that target very specific scenarios (milling, jamitons, bidirectional flows and other pedestrian dynamics, etc.) but very few can model the macroscopic regimes of these various distinct scenarios; compare, for example, figure 2 with [37, fig. 2] and with [48, fig. 8] or figure 3 with [20, fig 3].

On the other hand, we do acknowledge a drawback for working entirely in this discrete Voronoi topology. The rigid non-local framework of the Voronoi diagram (with topological changes at each time step) results in a model which is extremely difficult to analyse (even formally) in any precise mathematical framework. Indeed, the interesting collective behaviours are not in asymptotic parameter regimes and mean field (continuum) limits are intractable. While we certainly acknowledge this as a weakness from a modelling point of view, we nevertheless feel the merits of our motivation, its simple deterministic structure, its computational efficiency and its numerical predictions warrant the presentation here. Henceforth, our analysis of the VTP method is purely numerical; however, we stress that an additional advantage of the Voronoi setting is that it facilitates the computation of several observables to quantify certain generic collective behaviours. As we describe in §3.1 and §4, these include Voronoi-based notions of *clustering, pressure, percolation* and *queuing*. To our knowledge, these observables are new in the large collective behaviour literature, and can be applied not just to our VTP model, but to any *discrete time agent-based model* since these are independent of the dynamics, and can thus be computed on simulated or real-life data provided position and orientation information is available for every agent.

Our goal here is not to exhaust the possibilities of VTP nor tailor it to a specific biological or engineering system (see §5 for comments). Rather, we focus on two canonical scenarios: a point target and a narrow hallway. For the former, we work on the infinite plane and demonstrate interesting behaviours, including a novel *breathing* regime. For the latter, we consider a bidirectional flow in a hallway that exhibits lane formations and other interesting pedestrian dynamics.

In order to appreciate the VTP model, we supplement the article with a Github site.^1^ Here, one finds dynamic simulations for the runs discussed in this paper and many more. Specifically, the site presents a mixture of real-time simulations with adjustable parameters and recorded ones: many scenarios are explored in different spatial domains. One can download the code for further experimentation with VTP. Readers will also find there the written Appendix which includes various technical details and discussion.

With two controlling parameters and the inclusion of a target, it is difficult to fully exhaust the possible behaviours of our model. Thus, in the electronic supplementary material we present a complete numerical analysis for the simplest case: untargeted motions on two canonical compact manifolds without boundary, the flat torus and the 2-sphere. Here, we decompose the relevant phase diagram into five regimes; the reader is encouraged to consider the extreme regions of this diagram as ‘test’ cases to gain intuition on the dynamics obtained when repulsion dominates over alignment or vice versa (as the average density of agents varies). We also present in the electronic supplementary material simulations with point targets on both the flat torus and the 2-sphere.

## 2. The Voronoi topological perception model

The mathematics needed to present the VTP model are minimal: basically the notion of the Voronoi diagram is associated with a configuration of agents. While this does, however, introduce some notation, readers may simply focus on the following intuitive definitions. For completeness (and for those who wish to modify the GitHub code), we present the precise definitions.

Given a connected manifold Ω (prototypically a subspace of the Euclidean plane) with metric d , and distinct points ${𝐱}_{1},\ldots ,{𝐱}_{n}$ in Ω , the *Voronoi diagram* generated by ${𝐱}_{1},\ldots ,{𝐱}_{n}$ is the partition of Ω into the regions $V_{1},\ldots ,V_{n}$ where $V_{i}$ consists of all the points nearest ${𝐱}_{i}$ , precisely,


$$V_{i}=\{x\in \Omega :\;d(x,x_{i})\leq d(x,x_{j})\;\text{ for all }\;1\leq j\leq n\}.$$

The regions $V_{i}$ are called *Voronoi cells* and are always convex polygons in the sequel.

The Voronoi diagram’s geometric dual provides a natural structure to guide the inter-agent communication topology in our model.^2^ We will write i∼j to mean that ${𝐱}_{i}$ and ${𝐱}_{j}$ are adjacent in this dual, or equivalently, that their Voronoi cells $V_{i}$ and $V_{j}$ share an edge. For each i , we denote by *n*_*i*_ the number of Voronoi neighbours, $n_{i}=\#\{j:j∼i\}$ .

### 2.1. Governing equations

**Figure 4.**
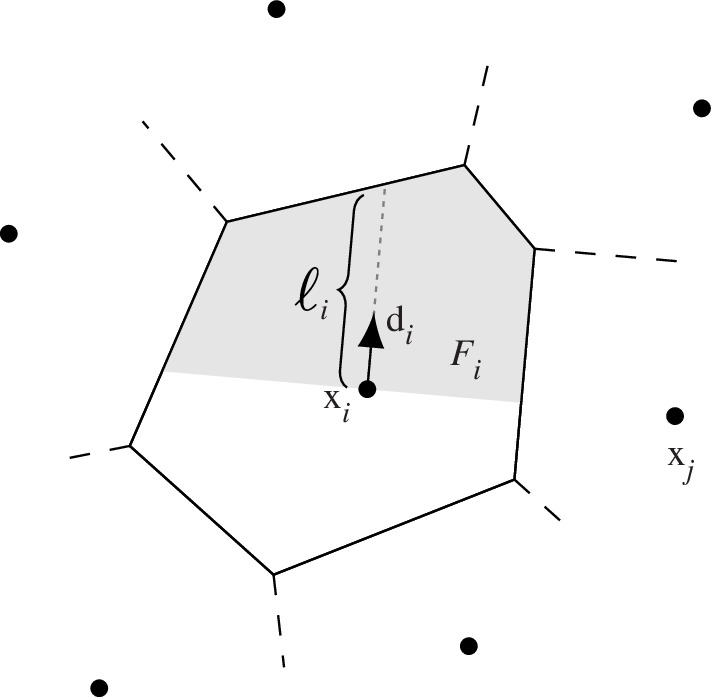
At each time step, the personal space of the *i*th agent is located at x*_i_* and its Voronoi-neighbouring agents (the position of a generic neighbour is labelled as x*_j_*). The desired direction vector d*_i_* associated with the *i*th agent determines the frontal area *F_i_* and frontal distance *l_i_* used to evaluate the personal-space speed *ρi* in equations (2.7) and (2.8) for Models I and II, respectively.

**Figure 5.**
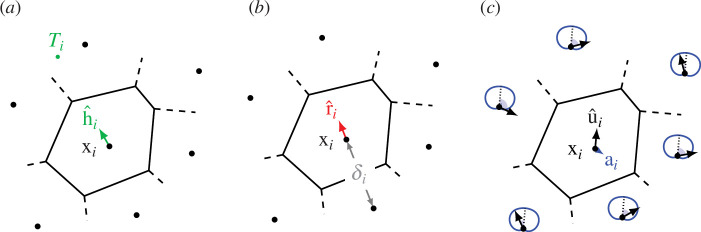
Schematic of the influences on a generic agent at time *t*. Here, we show one agent *i* at position x_*i*_ as well as its Voronoi cell and Voronoi neighbours whose positions are marked with black dots. We illustrate the three components that influence i ’s motion in the triptych above. Repulsion ${\hat{r}}_{i}$ and homing ${\hat{h}}_{i}$ are weighted with coefficients $\sigma_{i}=\sigma \,\left(\delta_{i}/L\right)$ and convex complement $1-\sigma_{i}=1-\sigma \,\left(\delta_{i}/L\right)$ , respectively, where *δ_i_* is the distance to i ’s nearest neighbour, as shown in (*b*). The relative weight of alignment **a**_*i*_ is given by the parameter *ν*. From the left, the diagrams are as follows. (*a*) Homing. Unit homing vector ${\hat{h}}_{i}$ points toward target *T_i_*, if it is non-empty and does not contain x*_i_*. (Here the target is shown as a dot but may be any region, in general.) (*b*) Repulsion. Repulsion vector always points away from nearest neighbor or domain boundary. The distance *δ_i_* to this nearest neighbor determines the relative weight of ${\hat{r}}_{i}$ and ${\hat{h}}_{i}$ . (*c*) Alignment. Alignment **a**_*i*_ is given by a weighted average of the orientations of Voronoi neighbors. The circularly wrapped weighting functions are indicated by the blue curves where the relative angle $\theta_{ij}$ (the angle between ${\hat{u}}_{i}$ and ${\hat{u}}_{j}$ ) marked with light blue sectors is the argument.

While the model was designed with numerous generalizations in mind, we present it here in its simplest form with two interpretations for the magnitude of personal space (Models I and II). Our model includes (i) the domain Ω , (ii) a set Λ of agent indices (which may change over time, as in §4), (iii) distinct positions ${𝐱}_{i}={𝐱}_{i}\,\left(t\right)\in \Omega$ for each i∈Λ , and (iv) closed (possibly empty) target regions $T_{i}\subset \Omega$ for each i∈Λ . Note that time here is arbitrary, and hence the discrete-time step is set to unity. Our model views the Voronoi diagram associated with the agent positions as fundamental to their perception (see figures 4 and 5).

At each time step t , we associate with the i th agent its *displacement vector* $u_{i}(t)=x_{i}(t)-x_{i}(t-1)$ . We denote by ${\hat{𝐮}}_{i}\,\left(t\right)$ the unit vector in the direction ${𝐮}_{i}\,\left(t\right)$ and refer to it as the i th agent’s *orientation vector* at time t . Since the time step is set to unity, we associate the magnitude of ${𝐮}_{i}\,\left(t\right)$ with the i th agent’s *speed* at time t . From the given initial positions and orientations, the trajectory is prescribed by a rule relating ${𝐮}_{i}\,\left(t+1\right)$ to the position and orientation vectors of the Voronoi-neighbouring agents at the previous time step t , namely, the system evolves according to an equation of the form


(2.1) $$x_{i}(t+1)=x_{i}(t)+f_{i}(X(t),U(t))\;\text{for all }i\in \Lambda ,$$

for functions ${𝐟}_{i}:\Omega^{n}\times {\left({\mathbb{R}}^{2}\right)}^{n}\to {\mathbb{R}}^{2}$ , where X and U are shorthand for $X\left(t\right)=\left({𝐱}_{i}\left(t\right):i\in \Lambda \right)$ and $U\left(t\right)=\left({𝐮}_{i}\left(t\right):i\in \Lambda \right)$ and n=#⁢Λ .

So, the behaviour of our model is then determined by the precise nature of ${𝐟}_{i}$ . Because we assume each agent has only local information, ${𝐟}_{i}$ will only depend on a narrow subset of agents—the Voronoi neighbours—at each instant, but their identities will change over time in general. The functions ${𝐟}_{i}$ are given by


(2.2) $$f_{i}(X,U)=\rho_{i}d_{i}\;,\;d_{i}=\frac{\sigma_{i}{\hat{r}}_{i}+\nu a_{i}+(1-\sigma_{i}){\hat{h}}_{i}}{1+\nu }.$$

Here, ${𝐝}_{i}$ is a weighted combination of three components ${\hat{𝐫}}_{i}$ , ${𝐚}_{i}$ , ${\hat{𝐡}}_{i}$ , *repulsion*, *alignment* and *homing*, respectively, with non-negative coefficients $\sigma_{i}$ , ν and $1-\sigma_{i}$ . Definitions of ${\hat{𝐫}}_{i}$ , ${𝐚}_{i}$ and ${\hat{𝐡}}_{i}$ are given in equations (2.3)–(2.5) and the weight $\sigma_{i}$ in equation (2.6). The coefficient ν is dimensionless and determines the strength of alignment compared with the combined homing–repulsion effect; ν is the first effective parameter of our model. We then scale by $\rho_{i}$ which depends on i ’s personal space and is defined later in equations (2.7) and (2.8). We emphasize that the components of ${𝐝}_{i}$ can be simply explained via the schematics in figure 5, which illustrates the heart and simplicity of the VTP model. The exact definitions of all these terms and the weight $\sigma_{i}$ are necessary for the specifics of the model, but we hope the additional mathematical notation involved does not obscure the core ideas.

Before presenting these details, we remark that equation (2.2) does not present a magnitude/direction decomposition, as ${𝐝}_{i}$ is not in general a unit vector. In a sense, ${𝐝}_{i}$ encapsulates the external influences on i while $\rho_{i}$ gives the speed scale i would like to achieve if allowed by ${𝐝}_{i}$ . Because of this, ${𝐟}_{i}$ can be small for two very different reasons: $\rho_{i}$ will be small when i has very little room to move and ${𝐝}_{i}$ will be small if repulsion, alignment and homing nearly cancel each other. However, $∥{𝐝}_{i}∥$ is on average bounded above by $1+\frac{1}{1+\nu }$ (cf. appendix), thus making ${𝐝}_{𝐢}$ a physically sensible direction of motion.

#### 2.1.1. Repulsion vector ${\hat{r}}_{i}$

The repulsion term ${\hat{𝐫}}_{i}$ (figure 5) is the straightforward collision-avoidance mechanism of *moving away from closest neighbour*; its use here is inspired by the work of Gonzalez *et al.* [51] in Voronoi energy minimization where experiments show that it facilitates the formation of homogeneous arrangements of agents.

Specifically, the *repulsion vectors* ${\hat{𝐫}}_{i}$ are given by


(2.3) $${\hat{r}}_{i}(X)=\frac{x_{i}-y_{i}}{\left\|x_{i}-y_{i}\right\|},$$

where ${𝐲}_{i}$ is the position of the ‘obstacle’ nearest ${𝐱}_{i}$ . Here, the word obstacle refers to the other agents and the domain boundary, if it exists. Precisely, ${𝐲}_{i}$ minimizes $\left\|x_{i}-y\right\|$ among 𝐲 in $\left\{{𝐱}_{j}:j\neq i\right\}\cup \partial \Omega$ . In the typical case, this is uniquely determined and we account for the edge cases by averaging.

We also define $\delta_{i}=\|x_{i}-y_{i}\|$ to be the unique distance from ${𝐱}_{i}$ to its nearest obstacle, as indicated in figure 5. The value $\delta_{i}$ will be used in the weighting coefficients (see §2.1.4) wherein its size is assessed via our second parameter L , the length scale within repulsion is active.

For many parameter ranges, there is a short-time oscillatory structure to ${\hat{𝐫}}_{i}$ resulting from Voronoi–neighbour connectivity changes (see [51] for more details). In these cases, the late-time animations show a ‘jittering’ in the individual agents’ directions. We do not see this as weakness in our model as agents on a small time scale may very well have a frenetic nature which averages out over large temporal and spatial scales.

#### 2.1.2. Alignment vector ${𝐚}_{i}$

Alignment is illustrated schematically in figure 5*c*. We define the *alignment vector* $a_{i}$ by the rescaled weighted average


(2.4) $$a_{i}=a_{i}(X,U)=\phi_{i}\cdot \frac{1}{n_{i}}\sum_{j∼i}g(\theta_{ij}){\hat{u}}_{j},$$

where, recall, *n*_*i*_ is the number of Voronoi neighbours of ${𝐱}_{i}$ and ${\hat{𝐮}}_{j}={𝐮}_{j}/∥{𝐮}_{j}∥$ is the orientation vector of agent j . Here, $\theta_{i\,j}=\arccos\left({\hat{𝐮}}_{i}\cdot {\hat{𝐮}}_{j}\right)$ is the angle between ${\hat{𝐮}}_{i}$ and ${\hat{𝐮}}_{j}$ , and g:[0,π]→[0,1] is a continuous non-increasing function with g⁢(0)=1 and g⁢(π)=0 . Thus, agent i considers the *orientation* of each of its neighbours and averages, favouring those whose direction is consistent with its own ( $\theta_{i\,j}$ near 0 ) and virtually ignoring those whose direction is opposed ( $\theta_{i\,j}$ close to π ). The role of the weighting g (more specifically its behaviour near 0 and π ) is crucial because it may tolerate more or less sheer in the flow depending on the modelled species. Put another way, the fact that agents can move in opposition to one another without much affecting this term manifests in interesting ways dynamically. For example, two opposing streams, if sufficiently sparse that repulsion is small, can pass through each other relatively easily with agents in each stream ignoring those in the other stream while reinforcing others in their own stream. However, an agent approaching a transversely moving group of others will be significantly deflected by it. We will see later two-way flow wherein non-jamming behaviours are much more accessible due to the weighting g . In the supplementary material, one also finds what we call *anti-cog* collective behaviour which exhibits very high sheer in the flow and does not occur without the fall-off of g at π .

The coefficient $\phi_{i}$ is simply $\phi_{i}\,\left(X\right)=n_{i}/6.$ To motivate this definition, we note that in any Voronoi diagram (in the torus, sphere, plane or planar region), a typical cell has at most six neighbouring cells (cf. appendix). So $\phi_{i}$ captures how ‘surrounded’ ${𝐱}_{i}$ is in the Voronoi topology. The effect of scaling the weighted average by $\phi_{i}$ is that agents with relatively few neighbours will be less strongly affected by this alignment interaction. Conversely, without $\phi_{i}$ , the alignment component of i would be crippled whenever i has many neighbours moving in the opposite direction. Overall, introducing $\phi_{i}$ mimics in outcome the improvement of *relative distance* brought by Motsch and Tadmor [4] over [3].

Noticing that alignment at time t depends on the neighbours at time t-1 , one may point out that since the previous time step t-1 , the neighbours j∼i may have changed. In particular, the neighbours of ${𝐱}_{i}\,\left(t\right)$ may include an agent j who did not neighbour agent i at t-1 (and was therefore invisible to them at the time); yet, according to equation (2.4), agent i is expected to have orientation information about that agent. We argue, however, that under reasonable assumptions, this does not in fact require agents to have any memory at all; the only assumption made is that every agent is able to infer the orientation of their neighbours from their current body geometry in an insignificant amount of time, e.g. by looking at their noses, tails, etc. Concretely, at time t , agent ${𝐱}_{i}\,\left(t\right)$ looks at all neighbours j∼i and gauges their orientations ${\hat{𝐮}}_{j}$ based on body geometry alone but does not need to infer any speed information $∥{𝐮}_{j}∥$ . Should the latter be the case, then agents would indeed need the memory of their neighbours’ positions ${𝐱}_{j}\,\left(t-1\right)$ at an earlier time. Thus, under our simple assumption on body geometry assessment, using unit length orientations as opposed to displacement vectors in equation (2.4) indeed makes our model ‘speed memoryless’, depending only on orientation features.

At last, we refer the reader to the appendix where a simple linearization of equation (2.4) before rescaling by $\phi_{i}$ shows that our alignment component incorporates three main terms: an inertial term aiming to preserve the heading of each agent i ; a ‘traditional’ unweighted average of the neighbours’ orientation; and a third ‘curling’ term containing the nonlinear influence of the neighbours j∼i onto i .

#### 2.1.3. Homing vector ${\hat{𝐡}}_{i}$

The homing term is shown for a simple-point target in figure 5*a*. This term simply points from ${𝐱}_{i}$ towards the target region $T_{i}$ . We define the target point ${𝐱}_{i}^{*}\in T_{i}$ by $∥{𝐱}_{i}^{*}-{𝐱}_{i}∥=\mathrm{dist}\left({𝐱}_{i},T_{i}\right).$ There is, in general, an issue of uniqueness here, but, in practice, this ambiguity is inconsequential because the set on which this definition is ambiguous has measure 0 in Ω . The *homing vector* ${\hat{𝐡}}_{i}$ is given by


(2.5) $${\hat{h}}_{i}(X)=\frac{x_{i}^{*}-x_{i}}{\left\|x_{i}^{*}-x_{i}\right\|}\;\text{for }x_{i}\notin T_{i}.$$

To account for the possibilities that ${𝐱}_{i}\in T_{i}$ or $T_{i}=\emptyset$ , we define ${\hat{𝐡}}_{i}$ to be 0 if ${𝐱}_{i}\in T_{i}$ or $T_{i}=\emptyset$ . Thus, ${\hat{𝐡}}_{i}$ is a unit vector or else the zero vector.

#### 2.1.4. Weighting coefficients $\sigma_{i}$

The repulsion ${\hat{𝐫}}_{i}$ and homing ${\hat{𝐡}}_{i}$ appear in equation (2.2) with weights $\sigma_{i}$ and $1-\sigma_{i}$ ; these are defined by introducing the length scale L and a *repulsion cut-off function* σ⁢(⋅) . We refer to L>0 as the *repulsive fall-off distance* that indicates the maximal distance over which a repulsive action is triggered, it can also be used to capture the size of the agents. Precisely, after recalling that $\delta_{i}$ is the distance from ${𝐱}_{i}$ to its nearest neighbour or boundary (figure 5), we define


(2.6) $$\sigma_{i}=\sigma (\delta_{i}/L),$$

where the function^3^ σ:[0,∞)→[0,1] is continuous at 0 , non-increasing and satisfies σ⁢(0)=1 and σ⁢(1)=0 . In this way, L is one of the two effective parameters of our model and captures the preferred radius of empty personal space of agents. Thus, we see that the convex combination $\sigma_{i}\,{\hat{𝐫}}_{i}+\left(1-\sigma_{i}\right)\,{\hat{𝐡}}_{i}$ facilitates the following behaviour: if ${𝐱}_{i}$ is at least a distance L from all obstacles, then full priority is given to target seeking via ${\hat{𝐡}}_{i}$ . On the other hand, as obstacles encroach on ${𝐱}_{i}$ at distances less than L , collision avoidance via ${\hat{𝐫}}_{i}$ progressively takes priority over target seeking.

#### 2.1.5. Personal-space speed

So far, we have constructed a direction vector ${𝐝}_{i}$ for the direction of movement at the t th time step. We must now scale its magnitude with scalar $\rho_{i}$ in equation (2.2) based upon: a speed limit (here taken to be unity); and the agents’ frontal personal space (based upon direction ${𝐝}_{i}$ ). Here, we present two models with two possible interpretations of the ‘magnitude’ of the personal space, both illustrated in figure 4. Model I is based on the area of the frontal personal space. Precisely, for ${𝐱}_{i},{𝐝}_{i}\in {\mathbb{R}}^{2}$ , define $H\,\left({𝐱}_{i},{𝐝}_{i}\right)=\left\{{𝐱}_{i}+𝐰\in {\mathbb{R}}^{2}:{𝐝}_{i}\cdot 𝐰\geq 0\right\}$ to be the half-plane with inward normal parallel to ${𝐝}_{i}$ whose boundary contains ${𝐱}_{i}$ . Then, define^4^


Fi=Fi(X,U)={12area(Vi∩H(xi,di))if di≠0,12area(Vi)if di=0,

where, as always, $V_{i}$ is the Voronoi cell containing ${𝐱}_{i}$ (see figure 4 for a depiction of $F_{i}$ ). To non-dimensionalize $F_{i}$ , we use the length scale L we have already introduced—the repulsive fall-off distance—and consider the quantity $\frac{F_{i}}{\pi \,L^{2}/2}$ , rescaling $F_{i}$ by the area of the semicircle of radius L . Finally, to obtain a step size from this quantity which is physically reasonable, we must enclose it in an increasing function that behaves like the identity near zero and goes to unity asymptotically so that agents attain maximum speed of 1 when there is nothing in their way. For this, we take the hyperbolic tangent. Thus for Model I, the coefficient $\rho_{i}$ is given by


(2.7) $$\text{Model I:}\;\rho_{i}=\rho_{i}(X,U)=\tanh\left(\frac{F_{i}}{\pi L^{2}/2}\right).$$

Model II follows the same reasoning but is based upon $\ell_{i}$ , the length of the segment starting at the position ${𝐱}_{i}$ in the direction ${𝐝}_{i}$ to the boundary of the Voronoi cell $V_{i}$ containing ${𝐱}_{i}$ (see figure 4). For Model II, the coefficient $\rho_{i}$ is given by


(2.8) $$\text{Model II:}\;\rho_{i}=\rho_{i}(X,U)=\tanh(\frac{\ell_{i}}{L}).$$

As an important point of clarification, the quantities $F_{i}$ and $\ell_{i}$ along with their visual representation (figure 4) do not aim to model a limited field of vision for the population. On the contrary, the VTP framework assumes that agents have a full $360^{\circ }$ awareness, $F_{i}$ and $\ell_{i}$ are just two different ways to gauge the size of one’s personal space once a direction ${𝐝}_{i}$ has been established. To conclude on the definition of the VTP model, we remark that equations (2.1–2.8) only effectively depend on the orientations $\left\{{\hat{𝐮}}_{i}\,\left(t\right)\right\}$ but not on the speeds $\left\{\|u_{i}(t)\|\right\}$ ; i.e. agents are ‘speed memoryless’ as they determine their speed at t+1 solely by gauging the geometry of their personal Voronoi space and by combining unitary directions.

#### 2.1.6. Summary of the parameters

To summarize, VTP involves two fundamental control parameters: the alignment coefficient ν and the repulsive fall-off distance L . The former is dimensionless and determines the relative strength of alignment ${𝐚}_{i}$ with respect to the repulsion–homing pair, while the latter is a length scale that specifies the preferred radius of an agent’s empty personal space. The number of agents n may be tuned but we confine our study to n between 500 and 1000. All the other ‘weights’ are directly determined by the local Voronoi geometry, modulo transition functions σ (for the weighting of repulsion with homing), g (for weighting neighbouring agent alignment) and tanh (for speed adjustment in $\rho_{i}$ ); for the former two, we made canonical choices (see footnote 3). We note, however, that these transition functions can be modified to encode constraints proper to specific populations; e.g. the canonical choice we made for g allows for (although does not enforce) an undisturbed percolation of agents as results show in §4.2, but a species that is highly sensitive to counterflow can be modelled using g⁢(π)≃1 . We note that there are two additional parameters which have been set to unity by rescaling: the time step and a characteristic speed intrinsic in our definition of $\rho_{i}$ .

## 3. Single-point target in the plane

### 3.1. Observables

To quantify our simulations in the various regimes, we consider comparable observables in addition to the angular momentum, namely, the median (relative) radius given by


$$r_{med}=r_{med}(X)=\underset{1\leq i\leq n}{\mathrm{median}}\;\|x_{i}-\bar{x}\|,$$

where $\bar{𝐱}$ is the centre of mass of the ${𝐱}_{i}$ and n=#⁢X . This gives a measure of the size of the swarm which is insensitive to outliers. We introduce a global *pressure* defined in terms of the Voronoi diagram, namely,


$$P(X)=\frac{1}{n}\sum_{i}\frac{1}{\left|V_{i}\right|},$$

where n=#⁢X and $\left|V_{i}\right|$ is the area of the Voronoi cell containing ${𝐱}_{i}\in X$ in the diagram generated by X . In the case that $\left|V_{i}\right|=\infty$ , it is understood that $1/\left|V_{i}\right|=0$ . This *mean reciprocal area* is analogous to pressure in the following way. A back-of-the-envelop calculation (see below) suggests that, under certain regularity assumptions, if the bounded parts of two Voronoi diagrams fill the same volume, then the denser configuration, i.e. the one with more generators, has the larger mean reciprocal area and this relationship is sublinear, being closest to linear when there are many more bounded than unbounded cells. Moreover, we have the following scaling relationship $P(rX)=\frac{1}{r^{d}}P(X)$ in ${\mathbb{R}}^{d}$ . So, we have an analogue of the familiar proportionality P∝n/V between pressure, number and total volume (even though we are in an unbounded domain).

The ‘back-of-the-envelop’ calculation suggested above is as follows. Let ${\left\{V_{i}\right\}}_{1\leq i\leq n}$ be a Voronoi diagram in ${\mathbb{R}}^{d}$ whose bounded part has total volume V . Without loss of generality, say ${\left\{V_{i}\right\}}_{i\leq n_{0}}$ are all and only the bounded cells for some $n_{0}<n$ . Suppose that the bounded cells are equi-distributed in the sense that $\left|V_{i}\right|=V/n_{0}$ for each $1\leq i\leq n_{0}$ . Of course, this assumption is almost impossibly restrictive but one can argue that the pressure is stable under small perturbations.^5^ The pressure is given by


$$P=\frac{1}{n}\sum_{i}\frac{1}{\left|V_{i}\right|}=\frac{1}{n}\sum_{i\leq n_{0}}\frac{1}{\left|V_{i}\right|}=\frac{1}{n}\sum_{i\leq n_{0}}\frac{n_{0}}{V}=\frac{n_{0}}{n}\frac{n_{0}}{V}.$$

If $n_{0}∼n-C\,n^{1/d}$ , as is typical. Then fixing V , we have


$$PV∼\frac{(n-Cn^{1/d}{)}^{2}}{n}=n-O(n^{1/d}),$$

where the error term $O\,\left(n^{1/d}\right)$ is positive.

### 3.2. Results

Since the domain ${\mathbb{R}}^{2}$ with a single-point target is invariant under scaling, one might be tempted to conclude our choice of the repulsive fall-off distance L is inconsequential.^6^ While this is not exactly the case, we set L=1 for our analysis of the single-point target and refer to the appendix for further explanation/justification. With L=1 fixed, we study empirically the long-term evolution of the system for different numbers of agents n and values of the alignment strength ν . We take as the initial state uniformly random positions within a square of area n/2 centred about the target point and unit velocities with uniformly random directions (the initial speed has no effect on the dynamics since the previous speed is forgotten at each step, cf. §2.1.5). The long-term dynamics are robust to the initial conditions; we chose a square simply because (pseudo)random points in a square are easily generated. The area of n/2 is comparable to the eventual size of the swarm (for a wide range of values of ν ) and so this choice shortens the transient. The choice here which most significantly affects the dynamics is having the initial configuration centred on the target. Even if this is not so, we have found the long-term behaviour to be robust but having the target point outside the initial swarm often results in transient regimes lasting hundreds or thousands of iterations. For both Models I and II, for small ν , the homing effect drives the swarm into a disc centred on the target and the velocities are uncorrelated. The equilibrium density of this disc is about where homing and repulsion are balanced and this depends on the shape of the fall-off function for repulsion. As exemplified in figure 3*c*, for very large ν , the swarm forms a rolling cluster that itself orbits the target point while individuals make periodic near passes to the target point (‘near’ relative to the rest of the swarm). Due to the strong alignment, agents are very nearly aligned at each fixed time.

The intermediate values of ν observe more interesting dynamics. First let us address Model II in which speed updates depend on the length $\ell_{i}$ ; recall equation (2.8). Increasing ν from the lower extreme, one sees an increase in the angular momentum (with respect to the centre of mass and to the target) achieved by the swarm (after an initial transient) as the velocities become more correlated. Enter the *pinwheel* regime shown in figure 3*a*. The agents occupy a disc whose centre averages near the target with roughly uniform density and rotate in the same direction about the target. Agents on the outer edge of the swarm tend to move faster than others, having relatively long distances $\ell_{i}$ ahead. Further increasing ν , the centre of the pinwheel becomes unstable and a cavity opens up, entering the *ring* regime shown in figure 3*b*. The rings form robustly after a typical transient of a few hundred iterations for sufficiently small ν , with the ring diameter increasing with ν for each fixed n . As previously mentioned, the ring regime gives way to the orbiting cluster regime, figure 3*c*, for large ν fixed; however, one can coax the swarm into still larger rings at greater values of ν by first lowering and then gradually increasing ν during the simulation. The stability of these large coerced rings is unclear.

**Figure 6.**
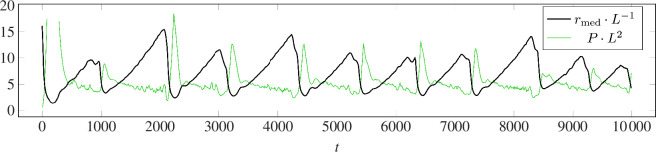
Example of the breathing regime observed under Model I for a single-point target in the plane. Here, there are *n* = 700 agents and the alignment strength is *ν* = 8. The curve (black) is the median radius of all agents (against time), i.e. the median distance to the centre of mass of the swarm. The secondary curve (green) is the Voronoi pressure. Each is non-dimensionalized with a suitable power of *L* (although here *L* = 1). The initial spike in pressure is clipped for space but the maximum is approximately 60. Click here to run a corresponding simulation.

Model I, in which speed depends on the area of the forward area $F_{i}$ , exhibits qualitatively different dynamics in the intermediate ν regime which we refer to as a *breathing regime*. Here, like Model II, the swarm forms a vortex about the target (after a short transient) and this vortex is filled for small ν and cavitated for larger ν . Unlike Model I, the size of the vortex is not constant in time. Rather, the cavity slowly grows over time between intermittent ‘inspiral collapses’; figure 6 shows these periodic collapses under the observables of median radius *r*_med_ and pressure P . The slow growth of the ring seems in part due to the fact that agents on the outer edge tend to have extremely large (or infinitely large) forward areas $F_{i}$ (see figure 4), and so move at nearly top speed, much faster than their inner neighbours. This speed difference causes the outermost agents to spiral further outward which in turn enlarges the Voronoi cells and the areas $F_{i}$ of their inner neighbours, propagating the speed increase inward. However, as the central cavity grows, so do the Voronoi cells of the innermost agents. The collapses occur when an agent on the inner edge of the ring deviates towards the centre (e.g. due to repulsion from an outer neighbour) and, having a large area $F_{i}$ ahead, deviates significantly. This effect propagates backward through alignment and the resulting enlargement in the Voronoi cells of trailing neighbours.

## 4. The bidirectional hallway

To showcase how our VTP framework naturally incorporates sources and sinks, we address its predictions in a narrow corridor Ω with two subpopulations looking to enter by each end and exit through the opposite one while interfering with each other throughout their crossing. Specifically; Ω is represented by a rectangle of width 1 and large enough length, the number of agents n=n⁢(t)=#⁢Λ⁢(t) varies since the index set $\Lambda (t)=\Lambda_{r}(t)\cup \Lambda_{l}(t)$ of all agents inside the hallway is no longer constant in time and consists of agents $X_{r}=\{x_{i}(t){\}}_{i\in \Lambda_{r}(t)}$ entering by its left edge and targeting its right edge (i.e. the entire right side represents the target $T_{i}$ for $i\in \Lambda_{r}$ ) together with the analogous subpopulation $X_{l}=\{x_{i}(t){\}}_{i\in \Lambda_{l}(t)}$ moving from right to left. Note that once an agent enters it can only exit through its corresponding target as all three other walls repel it. Details of the (stochastic) process governing the sources are discussed in the appendix.

### 4.1. Observables

To quantify the distinct behaviours exhibited by this bidirectional flow, we employ the following observables:

First the *polarization* proper to each subpopulation


$$S_{r,l}(U)=\frac{1}{\#\Lambda_{r,l}}\|\sum_{i\in \Lambda_{r,l}}{\hat{u}}_{i}\|.$$

This is a simple yet efficient order parameter widely used in the literature to measure heading consensus. Note that $0\leq S_{r,l}\leq 1$ and that we measure it for each subpopulation individually since the global polarization taken over i∈Λ is expected to be systematically small due to the symmetry of the scenario. We then measure *overall polarization* with $S=\frac{1}{2}(S_{r}+S_{l})$ .

Better suited to a bounded domain than the pressure P , we use the *clustering energy*


$$E(X)=\frac{n\cdot 18\sqrt{3}}{5|\Omega {|}^{2}}\sum_{i\in \Lambda }\int_{V_{i}}\|x-x_{i}{\|}^{2}dx,$$

to infer the overall spatial distribution of agents. As opposed to the Voronoi pressure from §3.1, this function measures the variances of ${\left\{V_{i}\right\}}_{i\in \Lambda }$ with respect to ${\left\{{𝐱}_{i}\right\}}_{i\in \Lambda }$ and thus, as agents are ‘better centred’ within their own Voronoi regions, the value of E decreases. Although this quantity arises frequently apropos of centroidal Voronoi tessellations (see [52]); to our knowledge, it has so far been absent in the vast literature of collective behaviour. Here, the constant $\frac{5|\Omega {|}^{2}}{n\cdot 18\sqrt{3}}$ represents the total variance of n regular hexagons tiling the domain Ω and is just a scaling allowing to compare values of E as n⁢(t) changes. Moreover, E⁢(X)≥1 for any spatial configuration X . The reader is referred to Gonzalez *et al.* [51] for more detail and properties of E .

To quantify *percolation*, i.e. the extent to which agents of a subpopulation entwine and venture into the other subpopulation, we define the *Voronoi interface length*


$$I(X)=\sum_{i\in \Lambda_{r};\;j\in \Lambda_{l}}|\partial V_{i}\cap \partial V_{j}|,$$

which is simply the total Euclidean length of the Voronoi boundaries separating the subpopulations.

Finally, a key structural behaviour that we wish to shed light on is *queuing*, namely, we wish to quantify a very specific type of ordered behaviour among agents of the *same subpopulation* who not only exhibit orientation consensus and certain spatial cohesion but also ‘align behind each other’ to form *lanes* oriented along the path towards their common target; this behaviour is anticipated in confined pedestrian scenarios (see [37,48]) but has also been observed for species in the wild (e.g. [53]). To this end, we define *queuing structures* $\Xi_{r}$ and $\Xi_{l}$ , weighted graphs which inherit part of the topology from the dual of the Voronoi diagram and also incorporate geometrical features about the current state ${\left({𝐱}_{i},{𝐮}_{i}\right)}_{i\in \Lambda_{r,l}}$ . Subsequently, an observable $Q(\Xi_{r,l})$ that measures their ‘queuing quality’ is defined.

For the purposes of this discussion, let DT⁢(X) denote the graph dual to the Voronoi diagram generated by X and let ${𝒟}_{r,l}$ its restrictions to the r,l subpopulations. Note that in general, ${𝒟}_{r}\neq \mathrm{DT}\,\left(X_{r}\right)$ .

Although any definition making up a reasonable queuing structure is highly subjective and open to debate, we postulate that the weighted graph $\Xi_{r}$ (and its analogous $\Xi_{l}$ ) needs to verify at least these four properties to intuitively showcase lane formations:

-
$\Xi_{r}$ is a subgraph of ${𝒟}_{r}$ ,
- each vertex of $\Xi_{r}$ has degree 1 or 2,
-
$\Xi_{r}$ is a forest, i.e. a (possibly disconnected) acyclic graph,
- if an edge $e_{i\,j}$ of $\Xi_{r}$ joins ${𝐱}_{i}$ and ${𝐱}_{j}$ , then its weight should be smallest in the case where the orientations ${\hat{𝐮}}_{i},{\hat{𝐮}}_{j}$ and homing vectors ${\hat{𝐡}}_{i},{\hat{𝐡}}_{j}$ all coincide.

The intuition behind these requirements is that after identifying each connected component of $\Xi_{r,l}$ with a *distinct lane*:

- two agents are contiguous in a lane only if they are from the same subpopulation and are Voronoi neighbours (and thus may interact via repulsion and alignment),
- a lane has no singleton vertices and is not ramified,
- a lane does not close on itself,
- we can locally quantify lane edge quality based on three simple geometrical elements; the orientations of the endpoint agents, their relative position and their homing. The smaller the weight, the more in sync the pair of agents is towards their common target region.

We refer to the appendix for details on the ad hoc construction of $\Xi_{r,l}$ we used in our work below and stress that there are, in general, many different graphs satisfying these postulates at any given time t . Results can thus fluctuate as variations of this construction are explored.

At last, let ${\left\{{ℒ}_{m}\right\}}_{m=1}^{M}$ represent the collection of M lanes composing $\Xi_{r}$ (i.e. its connected components), and then define the *queuing quality* observable $Q_{r}=Q\,\left(\Xi_{r}\right)$ by


$$Q_{r}=\frac{n}{\#vert(\Xi_{r})}\frac{1}{M}\sum_{m=1}^{M}\frac{weight(L_{m})}{{\left[\#edge(L_{m})\right]}^{2}},$$

where $\#\,\mathrm{vert}\,\left(\Xi_{r}\right)$ is the number of vertices of the whole queuing structure, $\Xi_{r}$ is the number of edges of the lane ${ℒ}_{m}$ and $\mathrm{weight}\,\left({ℒ}_{m}\right)$ is the total weight of (the edges of) the lane ${ℒ}_{m}$ . Indeed, this quantifies queuing according to four criteria: number of lanes M , overall number of edges of each lane (i.e. topological length of lanes), overall weight of each lane and number of agents belonging to $\Xi_{r}$ . As each one of these individual criteria improves while keeping the other three fixed, the value of $Q_{r}$ decreases. Thus, it is sensible to associate ‘good’ queuing with *ever lower values* of $Q_{r}.$ We define $\Xi_{l}$ and $Q_{l}=Q\,\left(\Xi_{l}\right)$ analogously; the *overall queuing quality* in the hallway at any given time is then captured using $Q=\frac{1}{2}(Q_{r}+Q_{l})$ .

In conclusion, besides the classical *polarization*, we have introduced observables to measure *clustering*, *percolation* and *queuing* that take advantage of and very naturally combine the (dual) Voronoi topology intrinsic to our model with elementary geometric features (position, angles and distances). We stress that these observables are parameterless and can be computed on any simulated or recorded data since they are independent of the model’s dynamics. This means that they can be used as ‘metrics’ to quantify differences between qualitative regimes, and, thus, can be used in optimizing a model’s parameter values to best fit observed data.

### 4.2. Results

Because n⁢(t) varies, its underlying degree of freedom is best represented by a constant quantity $L_{s}$ called the *source length scale* that accounts for the preferred interpersonal distance of agents entering the hallway. Specifically, if there is a half disc of radius $L_{s}$ centred somewhere on the entrance that is devoid of any agents, there is a large probability that a new agent will enter through that gap. Thus, the *smaller* $L_{s}$ is the *larger* the influx. Full detail on this stochastic entry process is presented in the appendix, but we remark that: (i) the inflow rate (in agents per time unit) is not constant and will diminish as the hallway becomes obstructed near the sources; and (ii) using $L_{s}$ to quantify inflow allows for a convenient comparison with the intrinsic repulsion length scale L .

Consequently, on top of our model’s parameters ν and L , the exogenous quantity $L_{s}$ also plays a crucial role in the dynamics. However, we claim that to qualitatively survey the emergent behaviours, one can categorize ν as either ‘weak’ or ‘strong’ and focus on the pair $\left(L,L_{s}\right)$ to draw a phase diagram since:

— weak alignment dynamics ( 0<ν≤1 ) are dominated by repulsion and homing, thus L and $L_{s}$ take precedence over ν ,
— strong alignment ( ν≥2 ) renders the influences of L and $L_{s}$ harder to predict. As will be presented below; larger ν values are characterized by the presence of vorticity due to non-negligible counterflow sheer.

We emphasize that, as opposed to the case $\Omega ={\mathbb{R}}^{2}$ from §3, the now present size and boundary effects make little to no qualitative difference between using Model I and Model II. In other words, as part of our observations, we encountered that having a non-negligible agent density on a restricted space produces very similar outcomes when agents base their speed upon personal forward area $F_{i}$ or on personal distance ahead $\ell_{i}$ , i.e. using equation (2.7) versus equation (2.8). For thoroughness, we included the results obtained with Model II in the appendix but the remainder of §4 will focus on Model I.

#### 4.2.1. Weak alignment

**Figure 7.**
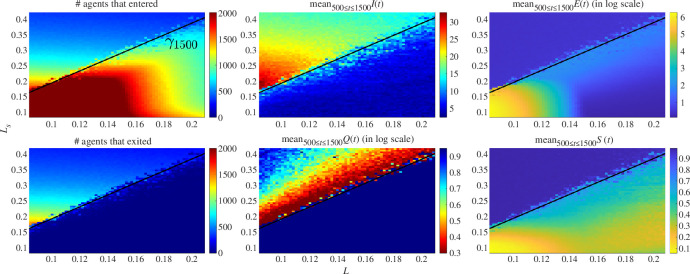
The (*L*, *L*_*s*_) phase diagram for Model I in the bidirectional corridor with weak alignment *ν* = 1: the length scale *L* for repulsion and the preferred empty length scale at the sources *L_s_* are at play (resolution of 65 × 65 points). (*Left*) The number of agents having entered and those having completed their crossing by the time *t*_max_ = 1500, a sharp bifurcation between full occlusion and sustained migration is marked by the line *γ* 1500: *L_s_* = 1.93*L* + 1.7·10^−3^ (*Centre and right*) The observables *I,Q,E* and *S* and (percolation, overall queuing quality, clustering and overall polarization) from §4.1 are averaged over the time tail *t* ∈ [500, 1500]. Remarkably, the same line *γ* 1500 shows a clear phase transition under each of our four observables. The region *L_S_* ≥ *γ* 1500 is characterized by the same number of entering and exiting agents as well as small *E* and large *I*; this translates to long-lasting sustained migrations with agents uniformly distributed. Moreover, the smooth increase of *Q* away from γ 1500 comes to further validate our postulates for the weighted graphs Ξ*_r,l_* as producing a sensible notion for queuing.

Figure 7 presents the phase diagram $\left(L,L_{s}\right)$ for ν=1 under several quantities. The maximal number of agents allowed to enter Ω was set to 1000 at each source and the dynamics evolved over t=1,…,1500 iterations. The four observables shown are averaged over the tail t∈[500,1500] to avoid any transient.

When looking at the number of agents that entered and exited by the time $t_{\text{max}}=1500$ , a clear bifurcation line $\gamma_{1500}$ emerges, where on one side the inflow is large enough ( $L_{s}$ small enough) to produce a complete occlusion of the hallway, and on the other side we see a full crossing of Ω since (almost) all agents having entered manage to exit through their respective target. The bifurcation line was numerically found to be


$$\gamma_{1500}:\;L_{s}=1.93\;L+1.7\cdot 10^{-3}$$

Remarkably, $\gamma_{1500}$ also signals a sharp transition under each of the four observables we defined in §4.1; clearly the non-trivial dynamics are found over $L_{s}\geq \gamma_{1500}$ where large polarization S and low clustering E indicate long-lasting and orderly migration uniformly distributed in space.

Furthermore, over the same region, percolation I decreases with $L_{s}$ while the overall queuing Q is optimal when closest to $\gamma_{1500}$ and increases again as we stray away from the bifurcation. The latter increase in Q is to be expected since our alignment components $\left\{{𝐚}_{i}\right\}$ (equation 2.4) only consider orientation and not position; thus according to this modelling choice, as the density in the hallway decreases (increase in $L_{s}$ ), agents are no longer prompt to press together and organize in lanes. Conversely, the smooth gradient of Q we observe above $\gamma_{1500}$ in figure 7 comes to validate our definitions for $\Xi_{r,l}$ and Q as being sensible constructions of what can intuitively be considered queuing.

Note that the measurements made for weak alignment are robust under the change of the random generator of the entry process.

At last, since our simulations are carried out in finite time and with finite maximal number of agents entering Ω , the bifurcation we measured may very well change with either quantity. Specifically, while the transition curve from complete occlusion to full migration can only move upwards in the phase diagram as we increase the time evolution of the dynamics; we conjecture that, as $t_{max}\to \infty$ and with an infinite number of agents at disposal, there exists a limiting curve $\gamma_{\infty }$ representing the ‘true’ critical bifurcation between eventual occlusion and sustained migration.

**Figure 8.**
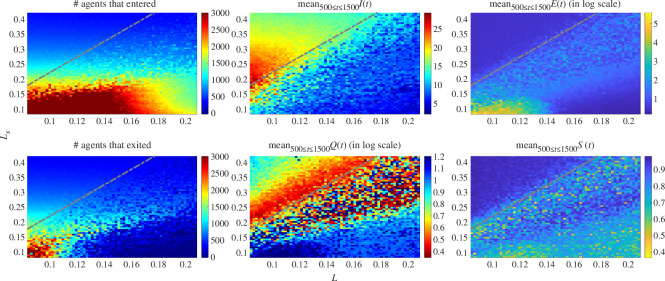
The (*L*, *L*_*s*_) phase diagram for Model I on the bidirectional hallway under strong alignment *ν* = 2: repulsive length scale *L* versus the preferred empty length scale at the sources *L*_*s*_ (resolution of 65 × 65 points). (*Left*) The number of agents having entered and those having completed their crossing by the time *t*_max_ = 1500 . (*Centre and right*) The percolation, queuing, clustering and polarization observables (*I,Q,E* and *S*) averaged over the time period *t* ∈ [500, 1500]. The transition between steady unobstructed migrations and full obstruction of the hallway is quite blurry as opposed to its sharp counterpart for the case *ν* = 1 shown in figure 7. The region of steady unobstructed migration (i.e. small *L* and large *L*_*s*_ ) that is qualitatively similar to its counterpart for *ν* = 1 is found above the dashed grey line *L*_*s*_ = 2.58*L* – 3.7 × 10^−2^; there the data are robust under change in the random generator of the agent’s entry. On the other hand, below the grey line the dynamics are rather unpredictable and showcase important vorticity.

We conclude on weak alignment with four specific regimes I–IV produced with L=0.0833 (the smallest L value shown in figures 7 and 8); their main characteristics are listed below and the animations of their time evolution are found in the Github site (*click on the regime labels below for the corresponding simulation*):

— Regime I. Here $L_{s}=0.1875$ is above the theoretical $\gamma_{\infty }$ and shows a large sustained percolation from the beginning, we are in the optimal queuing region (lowest Q values).
— Regime II. Very similar to Regime I in the long term with the difference that $L_{s}=0.1750$ being slightly smaller (larger influx) forces a turbulent transient before a long-lasting equilibrium with great queuing is established.
— Regime III. Here $L_{s}=0.1687$ is found between $\gamma_{1500}$ and $\gamma_{\infty }$ , meaning that a full occlusion eventually settles sometime after $t_{max}=1500$ . Nonetheless, for $t\leq t_{max}$ we see an interesting mixture of percolation, queuing and turbulence.
— Regime IV. $L_{s}\ll \gamma_{1500}$ produces a trivial regime where full occlusion settles in very fast and no interesting formations emerge.

Note that, by changing L we obtain similar qualitative behaviours as above provided $L_{s}$ is found in the corresponding regions, i.e. the behaviours remain comparable but with a more or less densely populated corridor.

#### 4.2.2. Strong alignment

Compared with weak alignment, the case ν≥2 exhibits dynamics that are not as predictable. While the two extreme cases, i.e. $L_{s}$ sufficiently large and sufficiently small, still produce steady unobstructed migrations and full obstructions, respectively; the transition from one to the other is quite blurry and significantly richer in dynamics thanks to the sheering effects capable of producing a large amounts of vorticity.

Figure 8 shows the $\left(L,L_{s}\right)$ phase diagram for ν=2 , where the maximal number of agents allowed to enter Ω was set to 1500 at each source and the dynamics evolved again over t=1,…,1500 . There a dashed grey line indicates where the blurry transition away from the steady migration region begins. We remark for the sake of thoroughness that the data were found to be robust under the random entry generator of agents for the region above the grey line but not below it.

Although lacking a well-established and robust region in the phase diagram, we have identified one persistent emergent behaviour famously known in the literature (see [54]):

— Regime V. Each subpopulation flows on respective sides of the corridor creating almost no percolation and an interface between them along the length of the hallway.

This regime is shown in figure 2 *b*; it reminds of a separated two-phase fluid flow along a pipe.

To show the reader other observed behaviours, the Github site also contains these regimes:

— Regime VI. With ν=2 , one subpopulation overcomes and manages to split the flow of the other in two; thus creating two interfaces along the length of the corridor. Here the $\left(L,L_{s}\right)$ values are in the blurry transition region showcased in figure 8.
— Regime VII. With ν=5 , vorticity completely dominates. Visually, this more resembles the growing and collapsing of mills in §3 than an ordered flow.

To conclude with the bidirectional corridor we remark that although the orientation of agents can be rather noisy when clustered together due to the nature of the repulsion components ${\hat{𝐫}}_{i}$ , the dynamics do average out over medium time scales and avoid the ‘freezing by heating’ effect known to disrupt all lane formation when noise is too great (see [55]).

## 5. Concluding remarks and future directions

We summarize our two main contributions:

— We present a model for the collective behaviour of agents based entirely on exploiting the local Voronoi topology (a natural notion of personal space) and geometry to synthesize three components—repulsion, homing and alignment. We show how this simple model can, with at most two controlling parameters, exhibit a variety of collective behaviours in different scenarios that can be visually explored in the Github site ^7^: rotating pinwheels, steady and *breathing* rings, different types of steady and ‘chaotic’ migrations across a hallway (in particular, formation of queues), highly polarized regimes with general velocity consensus, jamitons (i.e. stop-and-go waves) and full crystallization.
— We introduce and present several novel observables based entirely on the Voronoi diagram to quantify certain generic collective behaviours. These observables, decoupled from the dynamics, can be applied to any *discrete agent-based model* or to empirical data.

The numerical implementation of the VTP model is particularly simple in two dimensions. Indeed, simulations can be run and viewed in real time. The model and observables can easily be implemented in three dimensions as all the components have natural generalizations in three dimensions; the only caveat is that the Voronoi connectivity (Delaunay graph) is computationally expensive. Nevertheless, software is available.

While this is beyond the scope of the present work, a natural question to address is the extent one can use VTP to study the collective behaviour of a particular biological system. Moreover, it would be instructive to present a comparison of VTP with other models and a comparison with empirical data.^8^

Here, we remark that in addition to the controlling parameters ν and L , there are two unexplored degrees of freedom: (i) the structure of the function σ for repulsion weighting; and (ii) the function g for weighting neighbouring agent alignment. In both cases, we made canonical choices and verified the numerical stability with respect to these choices. However, one could tailor these, perhaps with data, to particular systems. For example, one could allow σ to eventually become negative, capturing *attraction/aggregation* at larger length scales. One could also explore the effects of the function ρ for speed adjustment.

We further emphasize that with minimal modifications the model can be applied to an extremely broad class of situations. With no modification whatsoever, the model as presented here allows for (i) any convex domain with or without boundary and (ii) arbitrarily many distinct classes of agents seeking distinct targets (each of which can be any subset of the domain). With minimal modification, our model can be made to (iii) include sources and sinks of agents (as in §4.2) and (iv) support non-convex domains so as to include obstacles (interior walls, pillars, …) in the environment. Such obstacles can be viewed as ‘holes’ or ‘inlets’ in the domain. The necessary modification to the model for such domains has to do with the Euclidean distance. A metric can be defined which is consistent with our assumptions for agents’ perception, and whose Voronoi diagram remains the natural fundamental structure upon which to construct VTP. While the modification is simple and natural, it does present certain computational difficulties in running simulations and this is the subject of current work. This raises the broader issue of constructing different metrics with which to build the Voronoi diagram. Voronoi diagrams in arbitrary metrics are much less well understood and computational methods involving them are lacking. Nonetheless, the question of determining the ‘right’ metric for a given set-up under VTP is intriguing.

Three other possible generalizations are as follows: (i) the alignment ${𝐚}_{i}$ of a population with higher situational awareness can be computed within a greater Voronoi radius, i.e. neighbours of neighbours, neighbours of neighbours of neighbours and so on. This can be implemented without a significant increase in computational complexity as one needs only compute powers of the already obtained adjacency matrix. Moreover, this property need not be the same among all agents. Indeed one might introduce variety among the agents both with respect to alignment and repulsion. (ii) Limited vision of the target regions can be modelled within the topological framework by allowing non-zero homing only when the target region is with some fixed number of Voronoi cells. We remark that the notion of topological radii naturally allows the integration of a component of attraction for aggregation in a more classical zone-based context. Specifically, alignment and attraction can act over concentric ‘layers’ having increasing Voronoi radii. (iii) The original VTP model as well as its possible extensions can be brought to heterogeneous crowds where agents act and respond differently to stimuli. An important example is when only a fraction of ‘active’ agents are mindful of their targets; very much like the effective leadership analysis performed in [30], the amount of target knowledge transferred to ‘passive’ agents can be studied to test the relevance of the VTP framework in the context of panic crowd dynamics.

## Data Availability

The code for all the simulations is available at [58]. Supplementary material is available online [59].

## Footnotes

^1^

https://jacktisdell.github.io/Voronoi-Topological-Perception

^2^
In the Euclidean metric, this dual graph is known as the Delaunay triangulation, see [49,50].

^3^
We take the repulsive cut-off function $\sigma (s)=\frac{z(1-s)}{z(s)+z(1-s)}$ where z(s)=exp⁡(−1/x) and alignment weighting function g(s)=σ(s/π) .

^4^
To motivate the $d_{i}=0$ case, we employ a probabilistic argument. The expected value of $V\cap H(x_{i},d_{i})$ for arbitrary $x_{i}$ and measurable set V over $d_{i}$ from a radially symmetric distribution is half the measure of V . The proof is given in the appendix.

^5^
Specifically, by first restricting to a sufficiently large closed ball including the bounded part of the Voronoi diagram and change, one can argue that for any ε small enough, there exists δ>0 such that if $\|x_{i}-x_{i}^{\prime }\|<\delta$ for each i and also $x_{i}^{\prime }$ is in the convex hull of the perturbed points if and only if $x_{i}$ belongs to the convex hull of the original points, then $(1+\varepsilon {)}^{-1}P\leq P^{\prime }\leq (1-\varepsilon {)}^{-1}P$ . The details are provided in the appendix.

^6^
Simulations on the VTP site for point targets on compact manifolds without boundary do vary L .

^7^

https://jacktisdell.github.io/Voronoi-Topological-Perception

^8^
For example, comparison of VTP against other agent-based models in the manner of [56] and quantitatively against empirical data as in [57] would clarify which settings VTP and our methods are most applicable.
